# Teaching-learning process of clinical skills using simulations - report of experience

**DOI:** 10.15694/mep.2019.000086.1

**Published:** 2019-04-15

**Authors:** Maria Helena Favarato, Murilo Moura Sarno, Lena Vania Carneiro Peres, Fernando Teles Arruda

**Affiliations:** 1 Universidade Municipal de São Caetano do Sul

**Keywords:** Undergraduate medical education, primary healthcare, empathy

## Abstract

This article was migrated. The article was marked as recommended.

Clinical skills training for medical students is critical so they can provide effective and safe care for patients and involves the development of technical skills, attitudes, and skills. The use of simulations allows the training of skills in a safe environment, with standardized and controlled learning opportunities. We report on the experience of simulation of medical care in basic care settings, home visits and consultations in the health unit, using professional actors who participate in the formative evaluation of the students, through the exposure of their perspectives and their feelings about care. Such a strategy allows the development of clinical skills in a humanized and patient-centered manner.

## Introduction

Clinical skills training for medical students is critical so they can provide effective and safe care to patients and involves the development of technical skills, attitudes, and skills. Clinical skills are defined as actions performed by health professionals directly involved in patient care, with impact on clinical outcomes in a measurable way ( Tolsgaard, 2013). This concept includes the association of cognitive aspects and technical and non-technical skills ( Tolsgaard, 2013).

The use of simulations allows the training of skills in a safe environment, with standardized and controlled learning opportunities ( Tolsgaard, 2013; Beigzadeh et al., 2016; Bokken et al., 2008). The closer the simulated environment to the actual practice environment, the easier it is for students to transpose knowledge into the field of practice with real patients. The presence of constant feedback leads to reflection, identification of critical points and improvement of practice.

Moreover, student-centered teaching-learning strategies have gained prominence. Our goal with this experience report is to discuss the integration between innovative student-centered teaching strategies and triggers for learning clinical skills based on simulations with professional actors through a curricular unit for the first and second years of the medical course.

## Experience Report

The Curricular Activity occurs from simulations of medical care in simulated scenarios. It uses professional actors in the role of patients or family/ companions, to interact with students. It explores cognitive, attitudinal and psychomotor content that underlies clinical practice. The stations are elaborated according to prevailing situations in the different life cycles and the proficiency profile of the student, according to the stage of the medical course in which they are. In the first Learning Cycle, the scenario is that of basic care, particularly the Family and Community Health units. In the first series, the competence profile to be developed implies key actions and performances in the three areas of competence, with emphasis on the area of attention to individual health needs and elaboration of therapeutic plans, through contact with patients in situations of simulated care in home visits or at the family health unit. In the second series, we have specific objectives of this activity clinical exploration of large syndromes and completion of the complete clinical examination, with recognition of normality patterns.

The student performs his/ her simulation, and, upon completing it, passes to the evaluation process of the experience. This process includes, in this order, a self-evaluation of its performance, simulated patient feedback on how it was felt during the service, feedback from the colleague who observed the attendance, and finally, problem-solving with the teacher, who retakes the actions performed, problematizes its rationale and significance for the student, making it possible to identify learning gaps. This moment generates a written production, which combines the patient’s medical history with a reflective narrative component about the experience. In a small group, students problematize the content of narratives and stories with the help of the facilitator and generate their learning questions that, after searching for scientific evidence, will be shared with the group.

As for the evaluative process, we have formative evaluation throughout the course, portfolio evaluation, assessment formats of the learning process and, annually, evaluation of simulated clinical practice, in a format similar to the practical test. Thus, the student’s movement towards the desired competences is identified; for the first year, in the area of individual attention, the main components are taken from the curricular matrix of competences:

A) Identifies Individual Health Needs

1. Perform clinical history: ethical relationship, bonding, clear guidelines, life context and biological, psychological and socioeconomic-cultural elements related to the health disease process.
2. Perform clinical examination: clarifies procedures, obtains consent, obtains anthropometric data, vital signs and conducts general clinical examination.
3. Formulate and prioritize problems: considering the personal, family, occupational, epidemiological, environmental, and other relevant contexts. Informs and explains the problems.

B) Builds and evaluates care plans

1. Develop care plan: pacts care actions with other professionals. It contemplates dimensions of self-care and health promotion. Search membership.
2. Accompany and evaluate plans of care: explains and guides, verifying understanding. Properly record the plan in the medical record.

For the second year, the following competencies are highlighted:

A) Identifies Individual Health Needs

1. Perform clinical history: investigates symptoms and signs, repercussions of the situation, habits, risk factors, conditions of vulnerability, related conditions and personal and family history. Recognizes major syndromes and is able to perform directed anamnesis.
2. Perform clinical examination: general clinical examination, cervical, pulmonary, cardiac, abdominal, dermatological and neurological and recognizes the normality patterns.
3. Formulate and prioritize problems: considering the personal, family, occupational, epidemiological, environmental, and other relevant contexts. Informs and explains the problems.

B) Builds and evaluates care plans

1. Develop care plan: pacts care actions with other professionals. It contemplates dimensions of self-care and health promotion. Search membership.
2. Accompany and evaluate plans of care: explains and guides, verifying understanding. Properly record the plan in the medical record.

## Discussion

The use of simulations in medical schools has been gaining ground in recent decades. The use of standardized patients began in the 1960s, with teachers and physicians unknown to students acting as patients ( Pate and Ricardo, 2016). This strategy allows identification of the student’s ability to identify problems and perform diagnostic and therapeutic maneuvers. However, patients’ emotional reactions, such as anxiety, fear and frustration, are important aspects of optimal clinical management and require specific reflection and training. Unlike standardized patients, simulated patients are those to whom a specific script is not provided, but a full text including symptoms, previous medical history, information on education, financial and social conditions, family and emotional aspects ( Beigzadeh et al., 2016; Pate and Ricardo, 2016). Based on this information, they can improvise in the contact with the students, giving their own emotional response, from the perspective of the patient, to the student ( Pate and Ricardo, 2016). Actors are encouraged to react according to the student’s situation and performance, as well as to disclose information only at the request of the students, who find themselves challenged to find unexpected and unforeseeable responses, requiring more students to develop empathy skills ( Pate and Ricardo, 2016). The use of professional actors as simulated patients is still restricted, both in Brazil and in the rest of the world ( Pate and Ricardo, 2016), and represents an innovation in this course that we describe. This aspect of medical training is very important and has been increasingly recognized as capable of modifying clinical outcomes ( Pate and Ricardo, 2016).

Unlike other simulation modalities, these scenarios allow for a more flexible approach and wider feedback than simply using checklists where the actions are categorized as correct and incorrect. This makes students more committed to bonding with their patients, being attentive to their mental and emotional states, and developing strategies to gain confidence and improve communication with their patients ( Beigzadeh et al., 2016; Bokken et al., 2008). Repeated contact with simulated situations with simulated patients decreases the students’ anxiety about their performance ( Beigzadeh et al., 2016; Bokken et al., 2008). With the development of this type of activity, receiving constant feedback, students from the beginning of their training are familiar with the fact that patients have feelings, impressions, experiences, beliefs and attitudes in relation to care that are very specific to each individual ( Beigzadeh et al., 2016) and this experience and reflection enable the student to be able to have empathy, capacity for dialogue and negotiation in the face of different situations.

Clinical skills are typically acquired in a complex, step-by-step development process with differentiated skills at each stage. The anagrama “RIME” describes the development of clinical expertise throughout the training of the student: Reporter (obtain information); Interpreter (analyzes and prioritizes patient’s problems); Management (elaborates care plan); Educator (reflects and educates others) ( Tolsgaard, 2013). These steps correlate with the Bloom taxonomy, starting with the knowledge of information, analysis, synthesis and evaluation ( Tolsgaard, 2013). The constant feedback in the simulations, from different sources - the colleague, the actor and the teacher - leads to self-reflection and, together with the work in a small group where situations are problematized, allows the identification of knowledge gaps, learning opportunity and the development of metacognition.

As for the possibility of evaluation by the colleague, we also find another rich opportunity, since the literature on strategies based on peers suggests that these are effective in the training of complex motor skills and have a positive impact on factors such as self-confidence and social aspects ( Tolsgaard, 2013).

The teacher’s role is to strengthen the student in the appropriation of his trajectory in the acquisition of the skills. Some strategies used in process evaluation and assistance feedback, which can be used in the simulations are: (1) the didactic question, whereby they incorporate the student’s reasoning in a process of reflection and learning. In this strategy, the questions asked by the teacher should be reflexive about the action performed, always based on the student’s performance in the simulation. Another possibility is to answer the student’s questions with other questions, so that the student himself identifies his / her knowledge gap and seeks to identify the solution based on his previous knowledge or later research in the literature. (2) Didactic empathy: through this the teacher understands the student’s perspective, which allows him to value his responses and actions, in addition to establishing a relationship with respect and trust. (3) Pedagogical silence as a tool for reflection. The teacher should become the person the student allows himself to question, rather than the one who will answer his or her questions ( Rodriguez-García and Medina-Moya, 2016).

With another look, the training of reflexive practice since graduation empowers and gives autonomy to the individual to have as habit to reflect on their situations of practical work, problematizing their experience and qualifying their daily professional practice ( Rodriguez-García and Medina-Moya, 2016).

As conclusion, we present an innovative methodology for the insertion of clinical skills in the undergraduate curriculum from the first year, with potential to favor the students’ critical and reflexive thinking. A differential of this activity is to count on professional actors who count their impressions on the service. The student also receives feedback from a fellow observer and the teacher in formative evaluation, being these materials for reflection and improvement of their practice, in different spheres, both technical and humanistic.

## Take Home Messages

• Clinical skills training is a critical point on medical education.
• Simulation-based training is a widely-used strategy, but using professional actors may enhance the reflexive component of the encounters.
• Early contact of the students with the simulated clinical practice may improve professional skills.

## Notes On Contributors

Maria Helena Favarato: ORCID: https://orcid.org/0000-0003-1039-216X. Specialist in Internal Medicine and Rheumatology. PhD in progress, “Education and Health”, University of São Paulo, School of Medicine.

Murilo Moura Sarno: ORCID: https://orcid.org/0000-0001-5429-0603. Specialist in family and community medicine. Master’s degree in course, ABC School of Medicine.

Lena Vânia Carneiro Peres: ORCID: https://orcid.org/0000-0003-1488-6458. PhD, São Paulo Federal University.

Fernando Teles Arruda - coordinator of medical school, Universidade Municipal de Sao Caetano. Specialist in Internal Medicine and Cardiology. Master’s degree, São Carlos Federal University.
