# Supplementary material for: Teaching-learning process of clinical skills using simulations - report of experience
Source: MedEdPublish (2016). 2019 Apr 15;8:86. [Version 1] doi: 10.15694/mep.2019.000086.1 (PMC10712630; doi:10.15694/mep.2019.000086.1)
Supplement: Peer review history

# Reviewer report: Reviewer response for version 1

**DOI:** 10.21956/mep.19806.r29805

**Published:** 2019-06-30

**Authors:** Debra Kiegaldie

**Affiliations:** 1 Holmesglen Institute & Healthscope Australia

This review has been migrated. The reviewer awarded 3 stars out of 5 This is a very interesting descriptive paper that would benefit from some minor restructuring. Firstly, I think three aspects need to be clearly defined within the introduction so that the reader knows exactly what it is that this paper is describing. So, what is the Curricular Activity, the Experience Report, and what is the nature of the clinical skills that is being included within this context. How and why are these being taught via simulation? There appears to be an assumption that the reader knows this background already. There is also reference to a plethora of clinical skills: technical skills, attitudes, skills, psycho-motor, non-technical skills, cognitive skills etc. This needs narrowing with clear definitions. Please note that the term non-technical skills may not be the best term to use as there is a view that communication skills are actually quite technical so I would generally try and avoid this as a term. In restructuring the paper, I would consider the following format. What did you do and why? How did you go about it? What happened? What would you do differently next time? Maybe a table might also be a better way of displaying the curricular matrix and how each component was evaluated? The discussion also focuses on simulation but this paper is just as much about a curriculum approach that incorporates simulation. Perhaps an equal weighting on these aspects might be useful. So the title might therefore be “Development of a curriculum matrix for teaching and learning of clinical skills using simulation: a report of our experience. I also acknowledge the authors for the inclusion of the simulated patient in providing feedback to learners. This paper has great potential with these changes in mind.

Reviewer Expertise:

NA

No decision status is available

# Reviewer report: Reviewer response for version 1

**DOI:** 10.21956/mep.19806.r29806

**Published:** 2019-04-22

**Authors:** Ramon Esperon-Hernandez

**Affiliations:** 1 University of Yucatan

This review has been migrated. The reviewer awarded 4 stars out of 5 Excellent experience developed around the use of simulation in clinical training.Some innovative aspects are identified, such as the use of professional actors and the competences established for the first and second year.It would be interesting to incorporate in a forthcoming paper the description of the educative processes that exist behind the training of the actors, the design of the scenarios and the instruments for measuring competences; as well as found results and if they have compared in some way the performance of the students with the different types of patients: standardized, simulated and / or robotics.

Reviewer Expertise:

NA

No decision status is available

# Reviewer report: Reviewer response for version 1

**DOI:** 10.21956/mep.19806.r29807

**Published:** 2019-04-21

**Authors:** P Ravi Shankar

**Affiliations:** 1 American International Medical University

This review has been migrated. The reviewer awarded 4 stars out of 5 I enjoyed reading this interesting article on the use of professional actors to strengthen learning of clinical skills during the first two years of the undergraduate medical course from a medical school in Brazil. While standardized and simulated patients are being increasingly used in medical schools there are some unique aspects to the program described. The process of reflection and feedback has been carefully considered. Self-reflection by students and retrospection on strengths and weaknesses is encouraged. This is supplemented by feedback from the patient, a colleague and the teacher. I would have liked a better description of how the program was developed and the various cases and simulations used during the first two years. I could not fully understand some of the competencies described by the authors. For example, first year, B builds and evaluates care plans, subsection 1, I did not understand the term ‘search membership’. The design of the program using professional actors allows for a more flexible approach to clinical scenarios as mentioned by the authors. All educators will find the article to be of interest.

Reviewer Expertise:

NA

No decision status is available

# Reviewer report: Reviewer response for version 1

**DOI:** 10.21956/mep.19806.r29804

**Published:** 2019-04-16

**Authors:** Gert Van Zyl

**Affiliations:** 1 University of the Free State

This review has been migrated. The reviewer awarded 4 stars out of 5 Excellent report on the use of simulation in an academic environment. It would have been worthwhile to know the resource environment of the specific institution - the experience is that simulation can play even a more important role in areas of resource limited academic environment. There is a limited reference to group work but it is deducted that this is more in a specific student cohort - the point to be made is the value of simulation in practicing team work as professionals for example in an resuscitation scenario. Continue on reporting on your experiences.

Reviewer Expertise:

NA

No decision status is available
